# Employing the X-Learner Algorithm to Evaluate the Intervention Effects of Physical Activity on Determinants of Elderly Mental Health

**DOI:** 10.3390/healthcare13111319

**Published:** 2025-06-02

**Authors:** Seungmo Kim, Taeyeon Oh

**Affiliations:** 1 Department of Sports and Health Sciences, Faculty of Arts and Social Sciences, Hong Kong Baptist University, Hong Kong, China; kimsm@hkbu.edu.hk; 2 Seoul AI School, aSSIST University, Seoul 03767, Republic of Korea

**Keywords:** physical activity, mental health, elderly, X-Learner algorithm

## Abstract

(1) Objectives: This study aimed to investigate the intervention effects of physical activity and sedentary behavior on the relationship between various influencing factors and mental health outcomes in the elderly. (2) Methods: Complied data collected from a nationwide survey conducted between 2013 and 2022 were analyzed using the X-Learner algorithm to explore these relationships. (3) Results: The findings indicate that engagement in both high- and moderate-intensity physical activities leads to statistically significant improvements in depression, suicidal ideation, and stress levels compared to non-participation. (4) Conclusions: The study emphasizes the essential role of physical activity in enhancing the mental health of the elderly in South Korea, demonstrating that high- and moderate-intensity exercise can effectively reduce depression, suicidal thoughts, and perceived stress. It also highlights the detrimental effects of prolonged sedentary behavior on the mental health of older adults.

## 1. Introduction

South Korea is experiencing a notable demographic shift, with the percentage of residents aged 65 and older rising from 15.1% in 2019 to an anticipated 20.0% by 2024 [1]. Comparably, in 2023, approximately 29.6% of Japan’s population and 21.65% of Hong Kong’s population were aged 65 or above [2,3]. The growing elderly population is expected to drive up demand for healthcare services, including hospitalizations and outpatient care, due to the high prevalence of chronic diseases and multimorbidity among older adults [4]. Consequently, healthcare costs are projected to rise substantially in the coming years.

This trend highlights the importance of examining both sedentary and non-sedentary activities among the elderly, as physical activity is frequently regarded as one of the key factors in enhancing their physical and mental health [5]. Understanding the effects of different activity levels can help in developing targeted strategies to improve overall well-being and potentially reduce healthcare expenses for this demographic in Korea. Promoting active lifestyles among older adults could lead to a reduction in the incidence of mental health issues such as depression and anxiety. Given the situation, the current study explored the relationships between the intensity of physical activity and the mental health of older adults.

### 1.1. Relationships Between Physical Activity Intensity and Mental Health Outcomes

Physical activity is often recognized as a vital component in promoting mental health and reducing the risk of mental health disorders [6,7]. Indeed, numerous studies have demonstrated the efficacy of exercise interventions for depression in older adults. Foundational meta-analyses confirmed that exercise significantly reduces depressive symptoms compared to control conditions in this population [8]. More recently, another meta-analysis demonstrated consistent positive effects of exercise interventions on depression in the elderly across numerous studies [9]. Similarly, Gallo and colleagues confirmed the robust association between physical activity and improved mental health outcomes in older populations, summarizing findings across multiple previous reviews [10].

The relationship between physical activity characteristics and mental health outcomes is intricate, and influenced by the type, intensity, and context of the activity. Fornaguera et al. [11] revealed that higher levels of physical activity could lead to significant mental health benefits. Doing more moderate-to-high-intensity physical exercise may reduce the risk of depression among adults, particularly in men and individuals aged 45 and older [12]. These findings showed that physical activity can improve mental health for certain groups, such as the elderly. Seminal work directly comparing exercise parameters found that high-intensity progressive resistance training was as effective as low-intensity training and standard care for treating clinical depression in older adults [13]. Investigating the dose–response relationship more broadly, a systematic review suggested that various amounts of physical activity can benefit mental health, although optimal parameters often require further clarification depending on the specific population and outcome [14]. A recent systematic review highlighted that different types of exercise, including aerobic and strength training, positively impacted mental health outcomes, even during challenging periods like the recent pandemic [15]. Furthermore, focusing on specific modalities popular among seniors, systematic reviews indicate that mind-body exercises like Tai Chi Chuan significantly improve mental health in elderly populations [16], and such approaches have also shown significant benefits for cognitive function, often correlating with improved overall well-being [17]. From a clinical perspective, the literature consistently supports integrating physical activity into mental health treatment plans, acknowledging its broad benefits [18]. Therefore, it is important to include suitable levels and types of exercise in programs designed to enhance mental health, representing a low-cost and accessible way to boost the well-being of older adults.

Sedentary behaviors, defined as activities involving low energy expenditure while sitting or resting, are a growing area of research due to their potential impact on health. These behaviors include both passive activities, such as sitting and listening to music or watching television, and intellectually active tasks, like writing or conducting research on a computer. Such activities occur in various contexts, including work, leisure, and transportation. Recent studies have highlighted the nuanced effects of sedentary behaviors, particularly among the elderly [19]. While it is generally accepted that sedentary behaviors can negatively impact physical and mental health, it is crucial to recognize that not all sedentary activities are harmful to older adults [20]. Intellectually engaging activities within this category can positively affect the elderly by enhancing cognitive performance and mental well-being, leading to better moods, reduced stress levels, and improved emotional health. On the other hand, non-sedentary behaviors, which involve any movement requiring energy expenditure, include light activities like walking or gardening and vigorous exercises such as running or swimming. These activities have received significant attention from researchers and are noted for their benefits to both physical and mental health, particularly among elderly adults [21].

In South Korea, as the country confronting the challenges of an aging society, understanding the impacts of both sedentary and non-sedentary behaviors is crucial for developing guidelines that promote health and well-being among older adults. In this context, the current study was designed to examine how various independent variables affect mental health, such as depression perception, suicidal thoughts, and perceived stress in the elderly across three groups: (a) those with sedentary behaviors, (b) those engaging in moderate-intensity physical activity, and (c) those involved in high-intensity physical activity.

### 1.2. Variables Affecting Mental Health

Mental health is a complex construct that can be influenced by various factors, such as demographics, physical health conditions, and lifestyle choices. The current study incorporated these factors, including demographic factors, health conditions, and health-related lifestyle choices, as input features in examining the relationship between physical activity intensity and mental health outcomes. Demographics like age [22,23] and gender [24,25] can play a significant role in mental health outcomes. For instance, females are more likely to experience depression and anxiety compared to males due to societal roles, hormonal factors, and caregiving responsibilities [24,26]. In addition, socioeconomic status can be an important factor in mental health, as individuals with lower incomes may often face higher levels of stress, financial instability, and limited access to healthcare, which can negatively impact mental health [23,27]. Health conditions can significantly influence mental health. Research has frequently shown that obesity is associated with increased rates of anxiety disorders [28]. However, both overweight and underweight individuals face a higher risk of developing mental health issues. For example, overweight individuals, particularly the elderly, are more likely to experience lower levels of psychological well-being, including reduced quality of life and life satisfaction [29]. Chronic diseases, such as diabetes, heart disease, and asthma, have a profound impact on mental health [30,31]. In fact, chronic diseases can influence mental health issues, while mental health conditions can also influence the progression of chronic diseases [22]. Healthy lifestyles, such as low alcohol consumption along with healthy diet habits, can influence mental health [32]. Particularly, alcohol consumption can influence mental health, as people with excessive alcohol consumption are likely to have more mental health issues like depression and anxiety [28,33].

In response to this context, the present study aimed to examine the intervention effects of physical activity and sedentary behavior on the relationship between influencing factors and the mental health outcomes of the elderly, using three indicators; depression [34], suicidal ideation [35], and perceived stress [36] among elderly individuals have been widely recognized in the literature as critical measures of mental health outcomes.

Research Question: What is the impact of varying levels of physical activity on the health outcomes of the elderly?

## 2. Methods

### 2.1. Data Source and Preprocessing

To address the research question, this study utilized data from the Korea National Health and Nutrition Examination Survey (KNHANES), a nationwide survey conducted jointly by the Ministry of Health and Welfare and the Korea Disease Control and Prevention Agency in South Korea. KNHANES has been conducted intermittently since 1998 and annually since 2007. This study compiled data from 2013 to 2022, as these years maintained a relatively consistent set of variables, ensuring comparability. The dataset includes the most recent publicly available data, with 2022 being the latest release.

From the total dataset spanning ten years, individuals aged 60 and above were screened, yielding 21,508 data points. However, given the tendency of older adults to provide incomplete or inconsistent responses, many entries contained missing values or responses such as “no response” or “do not know.” Data points containing such responses in variables used for analysis were removed, resulting in a final dataset of 7066 cases. The average age of individuals in the final dataset was 69.65 years (S.D. = 6.43), with 44.45% male and 55.55% female (See Table 1).

The dependent variables used to measure the mental health of older adults were selected based on commonly used indicators in previous mental health studies utilizing KNHANES. Specifically, three variables were used: perception of depression, suicidal ideation, and perceived stress. Perception of depression was measured by responses to the question, “Have you felt depressed for more than two consecutive weeks?” with a “yes” response indicating the presence of depressive symptoms. Suicidal ideation was determined based on responses to the question, “Have you ever felt the desire to commit suicide in the past year?” with affirmative responses categorized accordingly. Perceived stress was assessed using a five-point Likert scale response to the question, “Do you experience stress in your daily life?”. Individuals who answered “very much” or “a lot” were classified as experiencing high levels of stress (See Table 2).

Input features affecting mental health were selected based on commonly used factors in mental health research, including demographic characteristics, body mass index (BMI), health perception, the presence of major chronic diseases, and alcohol consumption. Demographic features included gender, age, educational attainment, income, household income, and occupational status. BMI was used alongside obesity classification (underweight, normal weight, overweight, obesity stage 1, obesity stage 2, and obesity stage 3). Health perception was assessed using a five-point Likert scale question, “Do you consider yourself healthy?”, with responses categorized as positive (“strongly agree,” “agree,” or “neutral”) or negative (“disagree” or “strongly disagree”). The presence of major chronic diseases was defined by the diagnosis of hypertension, hyperlipidemia, and diabetes. Alcohol consumption was categorized based on responses to the question “How often do you consume alcohol?” into ordinal categories reflecting increasing frequency. A detailed description of variables is provided in Table 1.

The key intervention feature in this study was physical activity among older adults, which was assessed using three indicators: high-intensity physical activity, moderate-intensity physical activity, and sedentary time per day. High-intensity physical activity was defined as activities such as running, jump rope, hiking, basketball, swimming, and badminton that cause significant shortness of breath or a rapid heart rate for at least 10 consecutive minutes. Individuals engaging in such activities at least once per week were classified as participating in high-intensity physical activity. Moderate-intensity physical activity was defined as activities such as brisk walking, light jogging, weight training (strength exercises), golf, dance sports, and Pilates that cause mild shortness of breath or a slight increase in heart rate for at least 10 consecutive minutes. The classification criteria were the same as for high-intensity activity, requiring at least one session per week. Lastly, sedentary time per day was treated as a continuous variable, measured by asking respondents to report the total time spent sitting per day, including activities such as sitting at a desk, socializing while seated, traveling in a vehicle, reading, writing, playing card games, watching television, playing video games, using the internet, and listening to music. Finally, the impact of sedentary behavior on mental health was examined by conducting separate analyses based on thresholds of 4, 6, 8, 10, and 12 h, determining whether engaging in more or less sedentary activity than each threshold influenced mental health outcomes (see Table 3).

### 2.2. Data Analysis

This study employs the X-Learner algorithm to estimate individual-level causal effects. The X-Learner is a meta-learning framework designed to estimate Conditional Average Treatment Effects (CATE) based on observational data, enabling the prediction of heterogeneous treatment effects at the individual level [37]. The method operates by first modeling the potential outcomes for both treated and control groups separately. Based on these estimates, pseudo-outcomes representing the potential treatment effects are calculated. These pseudo-outcomes are subsequently used to train models that produce the final individual-level treatment effect estimates. Observational data inherently lack counterfactual information. It is impossible to observe what would have happened to an individual if they had or had not received the treatment. The X-Learner framework provides a sophisticated mechanism to predict these unobserved potential outcomes. It also enables the comparison of the predicted outcomes. This approach facilitates a more precise estimation of individual causal effects.

Traditional statistical methods for treatment effect evaluation, such as regression analysis and propensity score matching (PSM), have been widely applied to estimate the Average Treatment Effect (ATE). These approaches primarily focus on assessing treatment effects at the aggregate level by comparing the average outcomes between treated and control groups. However, in contexts where significant heterogeneity exists across individuals or where treatment effects vary substantially at the individual level, such average-based methods may fail to capture these nuanced differences, leading to limitations in interpretability. Specifically, PSM may encounter challenges when sample sizes are small or when the distribution of propensity scores is heavily skewed, resulting in poor matching quality and biased effect estimates.

In contrast, the X-Learner offers structural advantages that address these limitations of conventional methods. By separately estimating the potential outcomes for treated and control groups and generating pseudo-outcomes, the X-Learner mitigates issues related to sample imbalance. Moreover, because it focuses on estimating CATE rather than ATE, the method facilitates the analysis of differential treatment effects across individuals, capturing variations in responses to the same treatment. This capability is particularly beneficial in research contexts characterized by high within group heterogeneity or when evaluating the impact of personalized interventions, enabling more detailed and practically meaningful interpretations compared to traditional statistical approaches.

Specifically, given that the mental health outcome variables (depression, suicidal ideation, stress) are binary, the estimated CATE represents the individual-level difference in the probability of experiencing the outcome attributable to the treatment condition. The average of these CATEs across the test dataset provides an estimate of the ATE on the probability scale, interpreted as a risk difference.

The analytical procedure of this study is as follows: First, base models are developed to predict potential outcomes for both the treated and control groups using the respective datasets. To enhance predictive accuracy, a range of machine learning and deep learning algorithms will be tested and compared. Specifically, traditional machine learning models such as Logistic Regression, Naïve Bayesian Classifier, Random Forest, Gradient Boosting Machines, and Support Vector Machine (SVM), as well as deep learning models like Deep Neural Networks (DNN) and Convolutional Neural Networks, will be evaluated. The model demonstrating the highest predictive performance will be selected as the final base learner. Using the selected model, potential outcomes for each individual in both groups will be estimated, and pseudo-outcomes will be computed accordingly. These pseudo-outcomes will then serve as the basis for training models that predict individual-level treatment effects. Ultimately, the CATE for each individual case will be estimated. Through this comprehensive process, the study aims to conduct a refined analysis of how the treatment influences individuals differently, providing granular insights into heterogeneous causal effects.

Building upon this framework, the present study applies the X-Learner method to examine the causal relationship between physical activity and mental health outcomes among older adults. The analysis focuses on three key behavioral variables: high-intensity physical activity, moderate-intensity physical activity, and sedentary time. For each variable, individuals are classified into treatment and control groups based on predefined thresholds. Specifically, participants who engage in high-intensity activities such as running, hiking, or swimming at least once per week are assigned to the treatment group, while those who do not meet this threshold are placed in the control group. A similar classification is applied to moderate-intensity activities, including brisk walking or light jogging. In the case of sedentary behavior, individuals whose daily sedentary time exceeds the sample average are categorized as the treatment group, while those below the average form the control group. This classification allows the study to capture both the positive effects of physical activity and the negative impacts of prolonged sedentary behavior.

The analytical procedure consists of three main stages. In the first stage, two separate outcome models are developed using deep neural networks. One model is trained in the treated group and the other in the control group. These models predict mental health outcomes, including perceived depression, suicidal ideation, and stress levels, based on a wide range of covariates such as demographic characteristics, body mass index, chronic disease status, alcohol consumption, and self-rated health.

In the second stage, individual-level treatment effects are estimated by generating counterfactual predictions. For each individual in the treated group, the model trained in the control group predicts their mental health outcomes under the hypothetical scenario of not engaging in the respective physical activity or spending less time in sedentary behavior. Conversely, for each individual in the control group, the model trained on the treated group predicts the counterfactual outcomes as if they had engaged in higher levels of physical activity or experienced more sedentary time. This process allows the study to estimate personalized treatment effects rather than relying solely on population averages.

In the third stage, a refinement model is applied to improve the precision of individual treatment effect estimates. A deep neural network is trained to smooth the estimates and adjust for potential confounding variables, enhancing the robustness and accuracy of the results. Additionally, weight adjustments based on propensity scores or other covariate balancing techniques are incorporated to reduce selection bias in the observational data. Through this structured process, the study aims to provide a detailed understanding of how physical activity and sedentary behavior influence mental health outcomes in older adults. The findings are expected to offer valuable insights for developing targeted health interventions and personalized policy recommendations.

Finally, this study conducted an effect size analysis to evaluate the magnitude and statistical significance of the treatment effect based on the estimated CATE. Specifically, the average treatment effect was calculated using the individual treatment effect estimates derived from the test dataset. The standard error of the estimated effect was computed by dividing the standard deviation of the individual treatment effects by the square root of the sample size in the test dataset. Subsequently, a 95 percent confidence interval was constructed using the critical value from the t distribution. The lower and upper bounds of the confidence interval were calculated by subtracting and adding the product of the t critical value and the standard error from the average treatment effect, respectively. This confidence interval was used to account for uncertainty in the estimated treatment effect and to assess whether the effect size was statistically significant. In addition, a *t*-test was performed to compute the *p*-value and further examine the statistical significance of the average treatment effect. All analyses were conducted in the Google Colab environment using Python 3.11.

### 2.3. Causal Inference Assumptions

The interpretation of the CATE estimated by the X-Learner relies on several key assumptions inherent to causal inference with observational data. Firstly, we assume ignorability (also known as conditional independence or no unmeasured confounding), which posits that the assignment to treatment (e.g., participating in high-intensity physical activity vs. not) is independent of the potential mental health outcomes, conditional on the observed covariates. To approximate this assumption, this study included a wide range of covariates detailed in Table 1, encompassing demographic characteristics, body mass index, chronic disease status, alcohol consumption, and self-rated health. Secondly, we assume Positivity (or Overlap), meaning that for any combination of these observed covariates, there is a non-zero probability of an individual being in either the treatment or the control group. Thirdly, the Stable Unit Treatment Value Assumption (SUTVA) is assumed, implying that an individual’s potential outcome is only affected by their own treatment status (their level of physical activity or sedentary behavior) and not by the treatment status of others, and that there are no different versions of the treatment leading to different outcomes. While these assumptions are necessary for causal interpretation, they may not be fully met in observational settings.

## 3. Results

Prior to analyzing the substantive treatment effects, this study conducted a model-selection process to identify the most appropriate predictive model. Specifically, each candidate model was evaluated based on its predictive performance in estimating the outcome variables using the input variables excluding the treatment variable. Three primary evaluation metrics were employed for model testing: prediction accuracy, Area Under the Curve (AUC), and F1-Score.

Prediction accuracy was used as a basic evaluation metric, representing the proportion of correct predictions made by the model relative to the observed values. This metric provided a general assessment of the model’s overall predictive performance. However, given that accuracy alone may not adequately capture issues related to data imbalance or the potential rarity of treatment effects, additional evaluation using AUC and F1-Score was performed.

AUC, which measures the area under the Receiver Operating Characteristic (ROC) curve, served as an important metric to comprehensively assess the model’s classification capability across different threshold levels. A higher AUC indicates better discrimination between cases with and without treatment effects. Considering the nature of this study, which requires distinguishing the magnitude and direction of treatment effects, AUC was regarded as a crucial indicator of model performance.

The F1-Score, calculated as the harmonic mean of precision and recall, was also utilized to evaluate model performance, particularly under conditions of data imbalance. Since rare cases of significant individual treatment effects may exist, the F1-Score was essential for assessing the model’s accuracy and reliability in predicting minority classes.

As presented in Table 4, each model exhibited varying strengths across the evaluation metrics. Ultimately, the DNN model was selected as the final model due to its consistently high and stable performance across all three evaluation criteria.

Following the selection of the final DNN model, a series of hyperparameter tuning experiments were conducted to enhance the predictive performance. The hyperparameters considered for tuning included the number of neurons in the hidden layers, activation functions, dropout rates, batch size, learning rate, and the number of training epochs. Iterative experiments were performed for each parameter to determine the optimal combination.

To optimize the architecture, various configurations ranging from two to four hidden layers were tested. For each layer, models with 64, 128, and 256 neurons were compared. The results indicated that a three-layer architecture with 256 neurons in the first layer, 128 neurons in the second layer, and 64 neurons in the third layer provided the most stable and robust learning performance. Rectified Linear Unit (ReLU) activation functions were applied to all hidden layers to capture non-linear relationships.

To prevent overfitting, dropout rates of 0.3, 0.5, and 0.7 were tested. A dropout rate of 0.5 achieved the best balance between performance and overfitting prevention and was adopted in the final model. Additionally, batch normalization was applied to each layer to reduce variance during training and improve convergence speed.

Experiments with batch sizes of 32, 64, and 128 and learning rates of 0.0005, 0.001, and 0.005 were also conducted. The combination of a batch size of 64 and a learning rate of 0.001 demonstrated the best performance in terms of predictive accuracy and training stability and was selected as the final hyperparameter setting. Furthermore, monitoring the model performance during training revealed signs of overfitting beyond 50 epochs. Therefore, the number of training epochs was set to 50.

Based on these step-by-step tuning experiments, the final DNN architecture and training conditions were established. The optimized model was then used to evaluate performance on the test dataset and to predict the treatment effects. The final model demonstrated a consistently high and stable performance not only in terms of prediction accuracy but also across AUC and F1-Score, confirming its suitability for estimating treatment effects.

Prior to measuring the CATE based on the finalized model, a SHAP (SHapley Additive exPlanations) feature importance analysis was conducted. SHAP is a method that fairly allocates and explains each feature’s contribution to individual predictions, providing an indication of how much each feature influenced the model’s final outcome. The analysis revealed that health perception was the most influential feature across all outcomes, namely depression, suicide ideation, and heavy stress (See Table 5). In the case of depression, additional key variables included sex, education level, and household income, with dyslipidemia emerging as a particularly important clinical factor. For suicide ideation, education level and household income were identified as major contributors. Finally, regarding heavy stress, the most influential factors were sex, occupation, and education level, in that order, with alcohol consumption habits also exerting a notable influence.

The results, summarized in Table 6, Table 7 and Table 8, present the estimated ATE based on the individual CATEs derived from the test dataset. These values represent the average difference in the probability of reporting perceived depression, suicidal ideation, or high stress levels associated with each treatment condition (high-intensity physical activity, moderate-intensity physical activity, or sedentary hours) compared to the respective control conditions. First, regarding high-intensity physical activity, individuals in the treatment group exhibited significantly greater improvements in all three mental health indicators—perceived depression, suicidal ideation, and stress levels—compared to the control group (See Table 6). Specifically, the intervention resulted in a 24% reduction in depressive symptoms, a 20% reduction in suicidal ideation, and a 17% reduction in perceived stress. These findings indicate that participation in high-intensity exercise leads to statistically significant improvements in mental health outcomes. Similarly, participation in moderate-intensity physical activity also resulted in statistically significant improvements in depression (3.7%), suicidal ideation (1.2%), and stress levels (4.2%) compared to non-participation. (See Table 7).

Finally, the effects of sedentary time on mental health outcomes were analyzed (See Table 8). Regarding depression, individuals who sat for less than 12 h per day exhibited a 9.4% reduction compared to those sitting for more than 12 h. When the threshold was lowered to 10 h, a 5.9% reduction was observed. While the reductions at 8 and 6 h were relatively modest, sitting for less than 4 h per day was associated with an approximately 4% decrease in depressive symptoms. A similar pattern was observed for suicidal ideation, where a notable reduction was evident when the threshold was set at 10 h; however, significant decreases were not observed at 6 or 8 h. For those sitting less than 4 h per day, a 1.2% reduction in suicidal ideation was identified. Likewise, stress levels demonstrated a similar trend. Individuals sitting for less than 12 h showed a 7.6% reduction compared to those sitting for longer periods, and those with sedentary time under 10 h exhibited an 8.5% reduction. Moreover, sitting for less than 4 h was associated with a substantial 4.8% decrease in stress levels. Taken together, these findings suggest that sedentary time exceeding 10 h per day has a substantially negative impact on mental health, whereas maintaining sedentary time under 4 h is markedly beneficial.

## 4. Discussion

The current study was conducted to emphasize the significant role of physical activity in enhancing the mental health of the elderly in South Korea, where the aging population is rapidly increasing. The study investigated the relationship between various influencing factors and mental health outcomes among the elderly, including depression, suicidal ideation, and perceived stress. It compared these outcomes among individuals based on their engagement in high-intensity physical activity versus those who do not, moderate-intensity physical activity versus those who do not, and extended sedentary periods versus those who do not.

The findings of this study align with the existing literature on the impact of physical activity and sedentary behavior on mental health in the elderly. Firstly, physical activity demonstrated an intervention effect on factors positively associated with mental health, indicating that higher levels of physical activity are linked to improved mental health outcomes. In this study, moderate-intensity activity contributed to positive mental health outcomes, suggesting that physical activity is crucial for reducing the risk of mental health disorders. Notably, high-intensity physical activity showed even greater improvements across all three mental health indicators. Fornaguera et al. [11] and Yang et al. [12] also found that moderate-to-vigorous exercise can decrease the risk of depression, particularly in older adults. These findings emphasize the importance of incorporating suitable exercise levels (moderate to vigorous) into mental health programs for the elderly, tailored to their specific conditions. Adopting a more vigorous approach to physical activity could provide a low-cost, effective, and powerful strategy for enhancing the well-being of older adults in aging societies like South Korea. Second, regarding the impact of sedentary behavior, the results indicated that individuals with below-average sedentary time showed significant improvements in depression and suicidal ideation compared to those with above-average sedentary time. While some sedentary behaviors, such as intellectually engaging tasks, may not negatively impact health [38], the findings of this study still suggest that lower levels of sedentary behavior are associated with reduced depression and suicidal ideation. This confirmed the adverse effects of prolonged inactivity on the mental health of the elderly [39].

Methodologically, this study contributes by introducing a new research direction through the application of the X-Learner approach utilizing DNN, extending beyond traditional statistical treatment effect models [40]. Conventional regression-based models fundamentally rely on the assumption of linearity. However, datasets like KNHANES, which undergo periodic sampling changes and contain inherent temporal dynamics, have limitations when the data are analyzed under the assumption of a cross-sectional data structure. In order to overcome the limitations, this study employed a data-driven AI analysis method, specifically DNN, to predict outcomes and estimate the treatment effects accordingly. This approach could enhance the accuracy and robustness of treatment effect estimation, offering valuable insights for future research utilizing large-scale health survey data.

While this study demonstrates significant mental health benefits associated with high-intensity physical activity among the elderly, careful consideration is warranted regarding its direct clinical application, particularly for individuals with pre-existing health conditions. Our sample included older adults with common chronic diseases such as hypertension, dyslipidemia, and diabetes, and the positive effects observed represent an average across this diverse group. Therefore, translating these findings into practice requires caution. Recommending high-intensity exercise regimens for elderly individuals, especially those with cardiovascular or other significant health issues, necessitates thorough individual assessment and potential medical clearance to ensure safety and appropriateness. The current findings underscore the potential benefits, but future research involving subgroup analyses focused on different health statuses is crucial. Such studies will help delineate differential effects and risks, paving the way for safer and more personalized exercise recommendations tailored to the specific health profiles of older adults.

The findings of this study underscore the significant benefits of physical activity for the mental health of the elderly, highlighting the importance of promoting active lifestyles among older adults to mitigate mental health issues and enhance overall well-being. However, several limitations should be considered for future studies. Firstly, although this study explored the relationship between various influencing factors and mental health outcomes alongside different levels of physical activity intensity, it focused on the overall relationship rather than isolating the individual effect of each factor. Therefore, future research should investigate the intervention effects of both sedentary and active behaviors on various demographic and lifestyle variables individually, in relation to mental health outcomes. By analyzing the impact on various subgroups based on the demographic and lifestyle conditions of elderly individuals, the findings can assist policymakers and healthcare providers in developing more comprehensive strategies or interventions tailored to support the mental health of elderly people from diverse backgrounds.

Second, while the KNHANES dataset offers large-scale and nationally representative data, it is based on self-reported physical activity and mental health conditions, which may introduce recall bias or social desirability bias. The study’s cross-sectional nature also limits the ability to draw causal inferences regarding the long-term impacts of physical activity and sedentary behavior on mental health outcomes. Therefore, longitudinal research should be considered to better establish causal relationships and capture changes over time.

Furthermore, while we controlled for a comprehensive set of observed features listed in Table 1, the possibility of unobserved confounding features influencing the relationship between physical activity and the elderly’s mental health outcomes due to the observational nature of the KNHANES data. Future research could employ sensitive analyses to assess the robustness of the estimated treatment effects to potential unmeasured features.

Finally, the evaluation of the predictive model’s performance and the subsequent treatment effect estimation was conducted using a held-out test set derived from the same KNHANES 2013–2022 dataset used for training. This constitutes an internal validation only. Consequently, the generalizability of the model and the robustness of the findings to truly external data—such as data from subsequent KNHANES waves beyond 2022, different national health surveys, or populations outside South Korea—have not been assessed. External validation is, therefore, a necessary next step for future research to confirm the stability and broader applicability of the reported treatment effects over time and across different contexts.

## 5. Conclusions

The current study emphasizes the vital importance of physical activity in enhancing the mental health of the elderly in South Korea. The results validate the positive impact of high- and moderate-intensity physical activity on mental health in reducing depression, suicidal ideation, and perceived stress among older adults. Furthermore, the findings also stress the detrimental effects of prolonged sedentary behavior on the elderly’s mental health. These findings provide valuable insights for the development of tailored strategies and interventions to support the mental well-being of older adults, ultimately promoting active lifestyles as a cost-effective approach to enhance the quality of life in aging societies.

## Figures and Tables

**Table 1:** Input features.

Features	Description
Sex [24,25]	0: male (3141, 44.45%) 1: female (3925, 55.55%)
Age [22,23]	Age (Mean = 69.65, S.D. = 6.43)
Education [27]	Education level 1: Elementary school or less (3403, 48.16%) 2: Middle school graduate (1541, 21.81%) 3: High school graduate (1215, 17.20%) 4: College degree or higher (907, 12.84%)
Income [27]	Income Quartile 1: Lower (1687, 23.87%) 2: Lower-middle (1752, 24.79%) 3: Upper-middle (1816, 25.70%) 4: Upper (1811, 25.63%)
Household Income [27]	Household income Qaurtile 1: Lower (2603, 36.84%) 2: Lower-middle (2069, 29.28%) 3: Upper-middle (1404, 19.87%) 4: Upper (990, 14.01%)
Occupation [27]	Occupation 1: Managers, professionals, and related workers (235, 3.33%) 2: Office workers (152, 2.15%) 3: Service and sales workers (535, 7.57%) 4: Skilled agricultural, forestry, and fishery workers (621, 8.79%) 5: Skilled manual and machine operators (481, 6.81%) 6: Unskilled laborers (1010, 14.29%) 7: Not in employment, including homemakers and students (4032, 57.06%)
BMI [28]	Body Mass index (Mean = 24.22, S.D. = 3.2)
Obesity Status [28]	Obesity Status 1: Underweight (182, 2.58%) 2: Normal Weight (2745, 38.85%) 3: Overweight (2060, 29.15%) 4: Mild obesity (1831, 25.91%) 5. Moderate obesity (220, 3.11%) 6. Severe obesity (28, 0.4%)
Health Perception [30,31]	Health Perception 0: not healthy (1854, 26.11%) 1: healthy (5221, 73.89%)
Hypertension [30,31]	0: No (3458, 48.94%) 1: Yes (3608, 48.94%)
Dyslipidemia [30,31]	0: No (4536, 64.19%) 1: Yes (2530, 35.81%)
Diabetes [30,31]	0: No (5656, 80.05%) 1: Yes (1410, 19.95%)
Alcohol Habits [32]	How often do you drink alcohol? 1: Have not consumed alcohol in the past year (3199, 45.27%) 2: Less than once a month (1114, 15.77%) 3: About once a month (543, 7.68%) 4: 2–4 times a month (934, 13.22%) 5: 2–3 times a week (728, 10.30%) 6: 4 or more times a week (548, 7.76%)

**Table 2:** Output features.

Variable	Description
Depression	Depressed Mood 0: No (6075, 85.98%) 1: Yes (991, 14.02%)
Suicidal Ideation	Suicidal ideation 0: No (6636, 93.91%) 1: Yes (430, 6.09%)
Stress	Feeling stress 0: No (5741, 81.25%) 1: Yes (1325, 18.75%)

**Table 3:** Intervention features.

Variable	Description
High-intensity PA	Vigorous physical activity 0: No (6854, 97%) 1: Yes (212, 3%)
Moderate PA	Moderate physical activity 0: No (6025, 85.27%) 1: Yes (1041, 14.73%)
Sedentary Hours	Sedentary hours less than 4 h (1398, 19.8%) 6 h (2706, 38.3%) 8 h (3078, 43.6%) 10 h (5318, 75.3%) 12 h (6083, 86.1%)

**Table 4:** Performance indexes for ML/DL models.

Model	Accuracy	AUC	F1-Score
Logistic Regression	0.864	0.679	0.801
Naive Bayes	0.833	0.678	0.825
Random Forest	0.855	0.648	0.806
SVM	0.864	0.589	0.801
XGBoost	0.849	0.646	0.813
LightGBM	0.852	0.646	0.804
DNN (Dense)	0.858	0.649	0.824
CNN (1D)	0.864	0.614	0.801

**Table 5:** SHAP feature importance on each output feature.

Features	Depression	Suicide Ideation	Heavy Stress
sex	0.0237	0.0046	0.0316
age	0.0118	0.0056	0.0136
education	0.0171	0.0104	0.0182
income	0.0111	0.0048	0.0071
household income	0.0151	0.0076	0.0094
occupation	0.0102	0.0072	0.0220
BMI	0.0069	0.0041	0.0059
obesity status	0.0057	0.0028	0.0116
health perception	0.0389	0.0239	0.0645
hypertension	0.0045	0.0041	0.0100
dyslipidemia	0.0103	0.0047	0.0099
diabetes	0.0084	0.0027	0.0061
alcohol habits	0.0058	0.0054	0.0102

**Table 6:** Treatment effect of high-intensity physical activity.

Mental Health Indicators	ATE	Lower Bound (95% CI)	Upper Bound (95% CI)	*p*-Value	Significant
Depression	0.246	0.242	0.249	0.001	True
Suicidal Ideation	0.203	0.202	0.205	0.001	True
Heavy Stress	0.172	0.170	0.175	0.001	True

**Table 7:** Treatment effect of moderate-intensity physical activity.

Mental Health Indicators	ATE	Lower Bound (95% CI)	Upper Bound (95% CI)	*p*-Value	Significant
Depression	0.037	0.031	0.044	0.001	True
Suicidal Ideation	0.012	0.010	0.014	0.001	True
Heavy Stress	0.042	0.036	0.049	0.001	True

**Table 8:** Treatment effect of sedentary hours.

Mental Health Indicators	Sedentary Hours	ATE	Lower Bound (95% CI)	Upper Bound (95% CI)	*p*-Value	Significant
Depression	4 h	0.040	0.034	0.045	0.001	TRUE
	6 h	0.002	0.001	0.002	0.001	TRUE
	8 h	0.002	0.001	0.003	0.007	TRUE
	10 h	0.059	0.051	0.068	0.001	TRUE
	12 h	0.094	0.087	0.101	0.001	TRUE
Suicidal Ideation	4 h	0.012	0.010	0.014	0.001	TRUE
	6 h	0.001	0.000	0.001	0.027	TRUE
	8 h	0.002	0.000	0.003	0.009	TRUE
	10 h	0.071	0.061	0.080	0.001	TRUE
	12 h	0.058	0.053	0.062	0.001	TRUE
Heavy Stress	4 h	0.048	0.042	0.053	0.001	TRUE
	6 h	0.006	0.004	0.007	0.001	TRUE
	8 h	0.007	0.004	0.010	0.001	TRUE
	10 h	0.085	0.074	0.095	0.001	TRUE
	12 h	0.076	0.070	0.082	0.001	TRUE

## Data Availability

The data presented in this study are available on request from the corresponding author. The data are publicly available through the KNHANES website (http://knhanes.cdc.go.kr, accessed on 14 March 2025).

## Abbreviations

The following abbreviations are used in this manuscript:

KNHANES	Korea National Health and Nutrition Examination Survey
BMI	Body Mass Index
CATE	Conditional Average Treatment Effects
PSM	Propensity Score Matching
ATE	Average Treatment Effect
SVM	Support Vector Machine
DNN	Deep Neural Networks
AUC	Area Under the Curve
ROC	Receiver Operating Characteristic
ReLu	Rectified Linear Unit

## References

[1] Ministry of the Interior and Safety (2024). The 2023 Resident Registration Population Is 51.33 Million, a Decrease of 110,000 Compared to the Previous Year. https://www.mois.go.kr/frt/bbs/type010/commonSelectBoardArticle.do;jsessionid=l5lsKvMK6B0kPxYlk8DOziw0.node10?bbsId=BBSMSTR_000000000008&nttId=106346.

[2] Statistics Bureau of Japan Current Population Estimates as of October 1, 2023. https://www.stat.go.jp/english/data/jinsui/2023np/index.html.

[3] TheGlobalEconomy.com Hong Kong Population Ages 65 and Above. https://www.theglobaleconomy.com/Hong-Kong/elderly_population/.

[4] Zhang D., Sit R., Wong C., Zou D., Mercer S., Johnston M., Wong S. (2020). Cohort profile: The prospective study on Chinese elderly with multimorbidity in primary care in Hong Kong. BMJ Open.

[5] Bustamante E.E., Brellenthin A.G., Brown D.R., O’Connor P.J. (2025). Up for Debate: Does Regular Physical Activity Really Improve Mental Health?. Med. Sci. Sports Exerc..

[6] Liu C., Liang X., Yang Y., Liu Y., Arbour-Nicitopoulos K.P., Sit C.H.P. (2024). Mechanisms linking physical activity with mental health in children and adolescents with neurodevelopmental disorders: A systematic review. Am. J. Prev. Med..

[7] White R.L., Vella S.A., Biddle S., Sutcliffe J.T., Guagliano J.M., Uddin R., Burgin A., Apostolopoulos M., Nguyen T., Young C. (2024). Physical activity and mental health: A systematic review and best-evidence synthesis of mediation and moderation studies. Int. J. Behav. Nutr. Phys. Act..

[8] Schuch F.B., Vancampfort D., Rosenbaum S., Richards J., Ward P.B., Stubbs B. (2016). Exercise for depression in older adults: A meta-analysis of randomized controlled trials. Am. J. Geriatr. Psychiatry.

[9] Zheng G., Chen C., Liu S., Zhang R., Huang Z., Liu Y., Li H., Chen Y., Li Y. (2020). Effects of exercise on depression in the elderly: A meta-analysis of randomized controlled trials. Int. J. Geriatr. Psychiatry.

[10] Gallo E., Pieri M., Daniele A., Gatti A., Masullo L., Sgadari A. (2023). Physical activity and exercise in relation to mental health outcomes in older people: An umbrella review. J. Affect. Disord..

[11] Fornaguera M., Dowda M., Pate R.R.R. (2024). Dose-response associations between physical activity and mental health among USA adolescents. Eur. J. Public Health.

[12] Yang D., Yang M.-C., Bai J., Ma Y., Yu C. (2022). Association between physical activity intensity and the risk for depression among adults from the National Health and Nutrition Examination Survey 2007–2018. Front. Aging Neurosci..

[13] Singh M.A.F., Stavrinos T.M., Scarbek Y., Galambos G., Liber C., Fiatarone Singh M.A. (2005). A randomized controlled trial of high versus low intensity weight training versus general practitioner care for clinical 3 depression in older adults. J. Gerontol. Ser. A Biol. Sci. Med. Sci..

[14] Zhang Z., Chen W. (2019). A Systematic Review of the Dose-Response Relationship Between Physical Activity and Mental Health. Int. J. Environ. Res. Public Health.

[15] Wang C., Tian Z., Luo Q. (2023). The impact of exercise on mental health during the COVID-19 pandemic: A systematic review and meta-analysis. Front. Public Health.

[16] Wu W.W., Liu T., Wang L.Q., Wang Z.J. (2015). Effects of Tai Chi Chuan on mental health of the elderly in China: A systematic review. Int. J. Nurs. Sci..

[17] Zou L., Wang C., Chen K., Liu Y., Zou Y., Zhang M., Cao C., Yan C. (2018). The Effect of Mind-Body Exercise on Cognitive Function in Older Adults: A Systematic Review and Meta-Analysis. J. Am. Med. Dir. Assoc..

[18] Faulkner G., Duncan M.J. (2017). Physical Activity and Mental Health.

[19] Li Q., Dong S., Liu X., Xu M., Song Y. (2024). Patterns of sedentary behaviors in older adults in Chinese care homes: A latent profile analysis. Innov. Aging.

[20] Wingood M., Bouldin E.D., Gell N. (2023). Sedentary behaviors and cognition: Does the type of behavior matter?. Innov. Aging.

[21] Yueh K.Y. (2024). Leisure activity participation among older adults: A review. Leis. Stud..

[22] Feng M.Y., Bi Y., Wang H.X., Pei J.J. (2023). Influence of chronic diseases on the occurrence of depression: A 13-year follow-up study from the Survey of Health, Ageing and Retirement in Europe. Psychiatry Res. Neuroimaging.

[23] Melchior M., Chastang J.F., Head J., Goldberg M., Zins M., Nabi H., Younès N. (2013). Socioeconomic position predicts long-term depression trajectory: A 13-year follow-up of the GAZEL cohort study. Mol. Psychiatry.

[24] Kim Y.J., Lee S.A. (2021). The relationship of lifestyle factors with the prevalence of major depressive disorder by ecological factors. Psychiatry Investig..

[25] Soldevila-Domenech N., Forero C.G., Alayo I., Capella J., Colom J., Malmusi D., Mompart A., Mortier P., Puértolas B., Sánchez N. (2021). Mental well-being of the general population: Direct and indirect effects of socioeconomic, relational and health factors. Qual. Life Res..

[26] Lim J.H. (2018). Regional differences of mental health status and associated factors: Based on the community health survey. Osong Public Health Res. Perspect..

[27] Tiwari S., Cerin E., Wilsgaard T., Løvsletten O., Grimsgaard S., Hopstock L.A., Schirmer H., Rosengren A., Kristoffersen K., Løchen M.L. (2024). Lifestyle factors as mediators of area-level socioeconomic differentials in mental health and cognitive function: The Tromsø Study. J. Epidemiol. Community Health.

[28] Rukavishnikov G.V., Rakitko A., Kasyanov E.D., Ilinsky V.V., Malyshko L.V., Neznanov N.G., Kibitov A.O., Mazo G.E. (2023). Association of depression and anxiety with somatic diseases: Negative lifestyle factors impact. S.S. Korsakov J. Neurol. Psychiatry.

[29] Rindler G.A., Gries A., Freidl W. (2023). Associations between Overweight, Obesity, and Mental Health: A Retrospective Study among European Adults Aged 50+. Frontiers in Public Health.

[30] Huang Y., Loux T., Huang X., Feng X. (2023). The relationship between chronic diseases and mental health: A cross-sectional study. Ment. Health Prev..

[31] Piane G.M., Smith T.C. (2014). Building an evidence base for the co-occurrence of chronic disease and psychiatric distress and impairment. Prev. Chronic Dis..

[32] Liutsko L., Leonov S., Pashenko A., Пoликанoва И. (2023). Frequency of Practicing of Different Types of Physical Activity, BMI and Tobacco/Alcohol Consumption Relationship: How They Are Associated with Our Physical and Mental Health in Different Ages, in Men and Women?. Preprints.

[33] Linardakis M., Papadaki A., Smpokos E., Micheli K., Vozikaki M., Philalithis A. (2015). Association of Behavioral Risk Factors for Chronic Diseases With Physical and Mental Health in European Adults Aged 50 Years or Older, 2004–2005. Prev. Chronic Dis..

[34] Zhong Q., Tian D. (2024). Mechanistic study of the effects of perceived community environment, high-intensity physical activity and public depressive status. Humanit. Soc. Sci. Commun.

[35] Fabiano N., Gupta A., Wong S., Tran J., Mohammad I.Y., Bal S., Fiedorowicz J.G., Firth J., Stubbs B., Vancampfort D. (2024). Physical Activity, Suicidal Ideation, Suicide Attempt and Death Among Individuals with Mental or Other Medical Disorders: A Systematic Review of Observational Studies. Neurosci. Biobehav. Rev.

[36] Smail E., Kaufmann C.N., King A.C., Espeland M.A., Anton S.D., Manini T.M. (2025). Effects of a physical activity intervention on perceived stress, fatigue, and depressive symptoms in older adults: A secondary analysis of the LIFE Study. J. Gerontol. Ser. A.

[37] Künzel S.R., Sekhon J.S., Bickel P.J., Yu B. (2019). Metalearners for estimating heterogeneous treatment effects using machine learning. Proc. Natl. Acad. Sci. USA.

[38] Cristi-Montero C., Hernández-Jaña S., Zavala-Crichton J.P., Tremblay M.S., Ortega F.B., Feter N., Mota J., Aguilar-Farias N., Ferrari G., Sadarangani K.P. (2023). Mentally active but not inactive sedentary behaviors are positively related to adolescents’ cognitive-academic achievements, a cross-sectional study—The cogni-action project. Ment. Health Phys. Act..

[39] Skelton D.A., Harvey J.A., Leask C.F., Scott J. (2023). Sedentary Behaviour and Ageing. Sedentary Behaviour Epidemiology.

[40] Smith B.I., Chimedza C., Bührmann J.H. (2023). Treatment Effect Performance of the X-Learner in the Presence of Confounding and Non-Linearity. Math. Comput. Appl..

